# Identifying the critical states and dynamic network biomarkers of cancers based on network entropy

**DOI:** 10.1186/s12967-022-03445-0

**Published:** 2022-06-06

**Authors:** Juntan Liu, Dandan Ding, Jiayuan Zhong, Rui Liu

**Affiliations:** 1 School of Mathematics, South China University of Technology, Guangzhou, 510640 China; 2 Pazhou Lab, Guangzhou, 510330 China; 3 School of Mathematics and Big Data, Foshan University, Foshan, 528000 China; 4 Department of Thoracic Surgery, Affiliated Cancer Hospital & Institute of Guangzhou Medical University, Guangzhou, 510095 China

**Keywords:** Critical state, Local network entropy (LNE), Pre-disease state, Critical transition, Dynamic network biomarker (DNB), Prognostic biomarker

## Abstract

**Background:**

There are sudden deterioration phenomena during the progression of many complex diseases, including most cancers; that is, the biological system may go through a critical transition from one stable state (the normal state) to another (the disease state). It is of great importance to predict this critical transition or the so-called pre-disease state so that patients can receive appropriate and timely medical care. In practice, however, this critical transition is usually difficult to identify due to the high nonlinearity and complexity of biological systems.

**Methods:**

In this study, we employed a model-free computational method, local network entropy (LNE), to identify the critical transition/pre-disease states of complex diseases. From a network perspective, this method effectively explores the key associations among biomolecules and captures their dynamic abnormalities.

**Results:**

Based on LNE, the pre-disease states of ten cancers were successfully detected. Two types of new prognostic biomarkers, optimistic LNE (O-LNE) and pessimistic LNE (P-LNE) biomarkers, were identified, enabling identification of the pre-disease state and evaluation of prognosis. In addition, LNE helps to find “dark genes” with nondifferential gene expression but differential LNE values.

**Conclusions:**

The proposed method effectively identified the critical transition states of complex diseases at the single-sample level. Our study not only identified the critical transition states of ten cancers but also provides two types of new prognostic biomarkers, O-LNE and P-LNE biomarkers, for further practical application. The method in this study therefore has great potential in personalized disease diagnosis.

**Supplementary Information:**

The online version contains supplementary material available at 10.1186/s12967-022-03445-0.

## Background

**Fig. 1:**
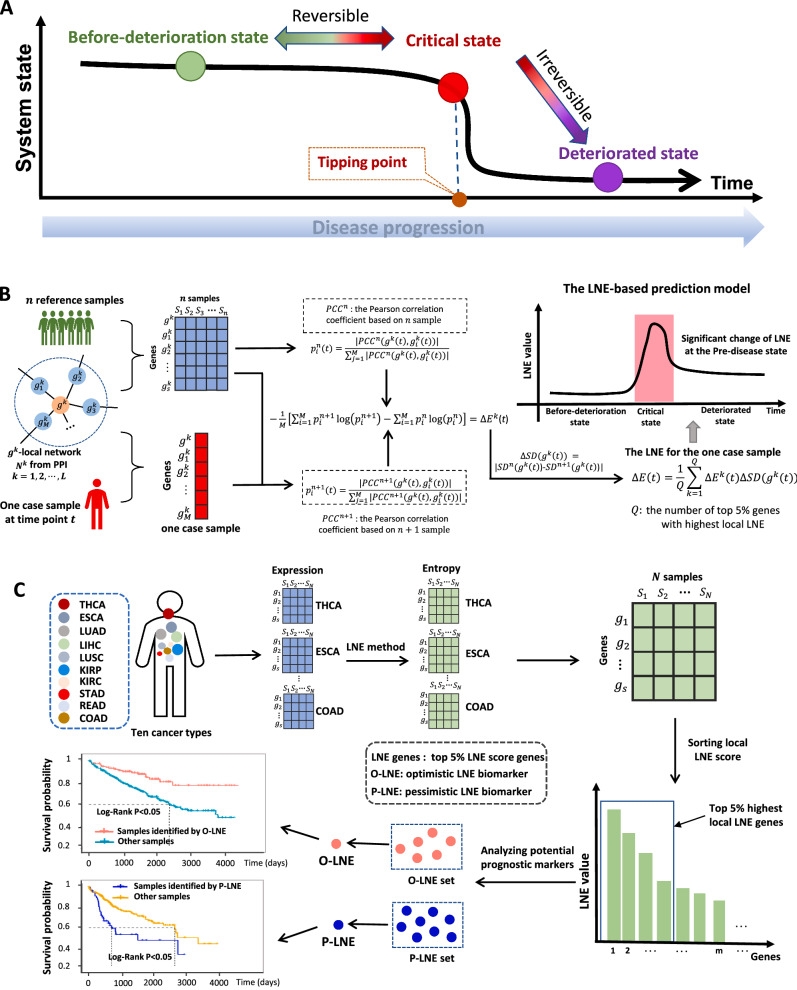
The overall design of this study. **A** Schematic diagram for disease progression of a complex disease in a subject. Regardless of specific differences in either biological processes or observed symptoms among diseases, the progression of illness can be generally divided into three stages or states, i.e., a relatively normal (before-deterioration) state, a pre-disease (critical) state, and a disease (deteriorated) state. A relatively normal state and pre-disease state are reversible state but a disease state is irreversible state. Thus, detection of the critical state is essential; **B** one-sample based local network entropy algorithm. Given a number of reference samples which can be derived from normal cohort, the LNE is calculated based on a single-sample from any individual. Specifically, both the reference samples and the to-be-determined single-sample are mapped to the existing PPI network or other reference network, which can be partitioned into local networks. For each local network centered on gene k, the local LNE score $\Delta E\left(t\right)$ is calculated; **C** for each cancer from the ten cancers and each individual from the cancer, calculating the LNE score of its each gene. After ranking the scores for all genes, the top 5% genes can be regarded as the LNE genes for the sample. LNE genes were further categorized to O-LNE and P-LNE biomarkers which enable significantly distinguish survival time between identified samples and unidentified samples

The progression of many complex diseases, such as cancer and diabetes, is not always smooth and occasionally change abruptly; that is, there exists a critical transition point at which the state switches from a relatively healthy state to a disease state or deterioration [1, 2]. Regardless of the specific differences in biological processes and/or observed symptoms among diseases, disease progression can be generally divided into three stages or states [1, 2], i.e., a relatively normal (before-deterioration) state, a pre-disease (critical) state, and a disease (deteriorated) state (Fig. 1A). The pre-disease state, which is the limit of the normal state, is unstable and reversible to the normal state with appropriate intervention, while the disease state is a stable state with high resilience and is almost irreversible [3–6]. Therefore, it is of great importance to detect early warning signs of the critical transition during disease progression and identify the pre-disease state so that timely medical care can be administered to prevent or at least postpone deterioration. However, it is not an easy task to identify such pre-disease states in biomedical practice due to both methodological and data limitations. First, during disease progression, the dynamics of biological systems generally involve a large number of molecules. Second, clinical data include issues of high dimensionality and noise perturbation, and sometimes, only few samples or one sample is available. The recently proposed dynamical network biomarker (DNB) theory shows a possible way of detecting the criticality of complex disease by regarding the progression of a disease as a high-dimensional nonlinear dynamic system and the critical transition as the state shift at the bifurcation point [1, 7, 8]. The DNB method and its modified versions have been applied in a variety of biomedical fields to successfully detect the pre-disease state of metabolic syndromes [9, 10], identify immune checkpoint blockades [11] and assess cell fate commitment [12]. However, the DNB theory is not suitable for the analysis of datasets with small sample sizes since it requires multiple samples at each time point to evaluate its three statistical conditions, which restricts its application for biological and clinical data. Therefore, there is still an urgent need for a suitable method that is capable of detecting the critical transition with only few samples or one sample.

**Fig. 2:**
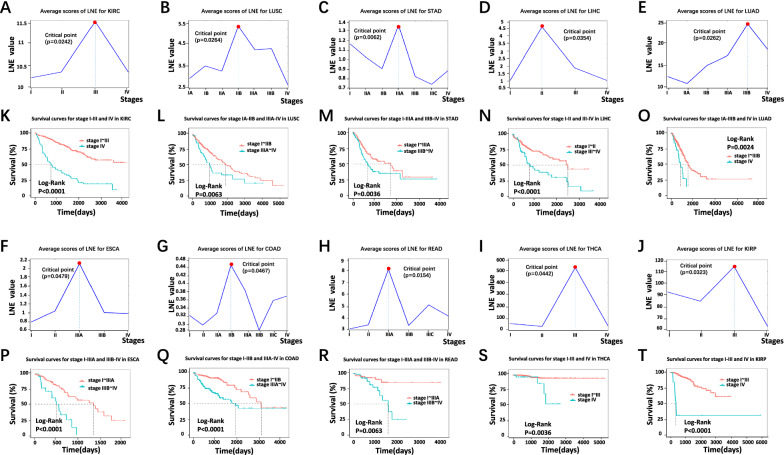
Identification of critical states preceding tumor deterioration in ten cancers: **A** KIRC; **B** LUSC; **C** STAD; **D** LIHC; **E** LUAD; **F** ESCA; **G** COAD; **H** READ; **I** THCA; **J** KIRP. Comparison of survival curves for samples taken before and after the critical state for ten cancers: **K** KIRC; **L** LUSC; **M** STAD; **N** LIHC; **O** LUAD; **P** ESCA; **Q** COAD; **R** READ; **S** THCA; **T** KIRP

In this study, a model-free computational method, local network entropy (LNE), was developed and employed to identify critical/pre-disease states and detect the early-warning signs of critical transitions. Specifically, for one given individual sample, the LNE score was calculated for each local biomolecular network (such as a protein–protein interaction (PPI) network) and then used to measure the statistical perturbation of the individual sample against a group of reference samples collected from a number of healthy/relatively healthy samples. The LNE method can characterize the dynamic difference between the normal and pre-disease states and further identify the pre-disease state during the progression of complex diseases. The LNE approach was applied for the datasets of ten different tumors from TCGA: kidney renal clear cell carcinoma (KIRC), lung squamous cell carcinoma (LUSC), stomach adenocarcinoma (STAD), liver hepatocellular carcinoma (LIHC), lung adenocarcinoma (LUAD), esophageal carcinoma (ESCA), colon adenocarcinoma (COAD), rectum adenocarcinoma (READ), thyroid carcinoma (THCA), and kidney renal papillary cell carcinoma (KIRP). For all ten tumor datasets, the critical states identified by LNE were present prior to severe disease deterioration; that is, a significant change in the LNE score was an early warning sign of the critical transition into disease deterioration. Specifically, the critical state for KIRC was identified in stage III disease before lymph node metastasis, that of LUSC was identified in stage IIB disease before lymph node metastasis, that of STAD was identified in stage IIIA disease before lymph node metastasis, and that of LIHC was identified in stage II disease before lymph node metastasis, and the patterns of other cancers are shown in Fig. 2.

In addition, we proposed a novel method (Fig. S1; the page S1 of Additional file 1) to classify LNE-sensitive genes into two types of biomarkers, i.e., optimistic LNE (O-LNE) and pessimistic LNE (P-LNE) biomarkers (Fig. 1C). Specifically, from the perspective of statistics, those samples identified as O-LNE biomarkers tended to have a relationship with good prognosis, while those identified as P-LNE biomarkers usually showed a trend towards correlation with poor prognosis. These biomarkers may also play important roles in disease deterioration. For instance, *CLIP4* is involved in regulating the expression of several tumor-associated genes, and its expression is considered to stimulate tumor metastasis [13]; for KIRC, gene *CLIP4* was identified as an O-LNE biomarker. For LUSC, gene *FGF11* was identified as an O-LNE biomarker, and *FGF11* may be involved in the stabilization of capillary-like tube structures associated with angiogenesis and may act as a modulator of hypoxia-induced pathological processes such as tumorigenesis [14]. For STAD, gene *ACE2* was identified as a P-LNE biomarker, and *ACE2* could affect macrophage expression of tumor necrosis factor (TNF-α) [15]. For LIHC, gene *TTK* was identified as a P-LNE biomarker, and *TTK* alone or in combination with other therapeutics might be able to selectively kill tumor cells [16]. Furthermore, in the analysis of these cancer datasets, the proposed method identified some “dark genes”, which showed nondifferential gene expression but differential LNE values.

## Methods

### Theoretical background

According to our recently proposed DNB theory [1, 2], disease progression can be generally divided into three stages or states, i.e., a relatively normal (before-deterioration) state, a pre-disease (critical) state, and a disease (deteriorated) state. The normal state is a stable state with high resilience and robustness to perturbation. The pre-disease state, which is the limit of the normal state, is unstable and reversible to the normal state with appropriate intervention. The disease state, which is also a stable state with high resilience, is almost irreversible [3–6]. Moreover, when a complex system is near the critical point, among all observed variables, there exists a dominant group defined as DNB biomolecules that satisfy the following three conditions based on the observed data [1]:

- The correlation (PCC_in_) between any pair of members in the DNB group rapidly increases;
- The correlation (PCC_out_) between one member of the DNB group and any other non-DNB member rapidly decreases;
- The standard deviation (SD_in_) or coefficient of variation for any member in the DNB group drastically increases.

All three of the above conditions are necessary for phase transitions, and can also be approximately stated as follows: the appearance of a strongly fluctuating and highly correlated group of features/variables implies imminent transition into the disease state. Therefore, we use these three conditions to quantify the tipping point as early-warning signs of the disease, and then the identified dominant group of biomolecules consists of DNB members. These three conditions are the theoretical basis of DNB theory and have been applied to a number of analyses of disease progression and biological processes to predict critical states [2, 5].

### Algorithm to identify the tipping point based on LNE

The LNE method uses reference samples (samples from normal cells that are regarded as the background and represent healthy or relatively healthy cells) and only one disease sample to identify the tipping point with the following algorithm (Fig. 1B). Specifically, inspired by the previous study [17], the term "entropy" proposed here aims to characterize the statistical perturbation brought by each individual sample against a group of given reference samples.

[Step 1] The first step is to form a global network ${\text{N}}^{G}$ by mapping the genes to a protein–protein interaction (PPI) network downloaded from STRING (http://string-db.org) [18], which contains the interactions of the selected genes with a confidence level of 0.800. Isolated nodes without any links to other nodes are discarded. The PPI network is identical to a template network for each individual sample.

[Step 2] The second step is to map TCGA data to the global network ${\text{N}}^{G}$. Data for ten cancers from the TCGA database are downloaded, and then, the gene expression data are mapped to the global network ${\text{N}}^{G}$ generated in the prior step.

[Step 3] The third step is to calculate the local network entropy for each gene. For each gene $g^{k}$, its local network ${\text{N}}^{k}$(k = 1, 2, …, L) is extracted from the global network ${\text{N}}^{G}$(Fig. 1B), where $\left\{ {{\text{g}}_{1}^{k} , \ldots ,{\text{g}}_{M}^{k} } \right\}$ are the 1st-order neighbors of $g^{k}$. *L* denotes the number of the local network. Then, the local entropy ${\text{E}}^{n} (k,t)$ is calculated based on n reference samples. The formula for calculating local entropy $E^{n} (k,t)$ is

1 $$ E^{n} (k,t) = - \frac{1}{M}\sum\limits_{i = 1}^{M} {p_{i}^{n} } (t)\log p_{i}^{n} (t), $$

with

2 $$ p_{i}^{n} (t) = \frac{{\left| {{\text{PCC}}^{n} \left( {g_{i}^{k} (t),g^{k} (t)} \right)} \right|}}{{\sum\limits_{j = 1}^{M} {\left| {{\text{PCC}}^{n} \left( {g_{j}^{k} (t),g^{k} (t)} \right)} \right|} }}, $$

where the constant *M* denotes the number of neighbors in the local network ${\text{N}}^{k}$ and ${\text{PCC}}^{n} \left( {g_{i}^{k} (t),g^{k} (t)} \right)$ denotes the Pearson correlation coefficient between the center gene ${\text{g}}^{k}$ and a neighbor ${\text{g}}_{i}^{k}$ based on *n* reference samples at time point *t*.

[Step 4] The fourth step is to calculate the differential entropy $\Delta E(k,t)$. $E^{n + 1} (k,t)$ is calculated based on *n* + 1 samples, mixing a single sample from an individual with *n* reference samples at time point *t*, i.e.,

3 $$ E^{n + 1} (k,t) = - \frac{1}{M}\sum\limits_{i = 1}^{M} {p_{i}^{n + 1} } (t)\log p_{i}^{n + 1} (t) $$

Then, the differential entropy $\Delta E^{k} (t)$ is calculated as

4 $$ \Delta E^{k} (t) = \left| {E^{n + 1} (k,t) - E^{n} (k,t)} \right|, $$

and

5 $$ \Delta SD\left( {g^{k} (t)} \right) = \left| {SD^{n} \left( {g^{k} (t)} \right) - SD^{n + 1} \left( {g^{k} (t)} \right)} \right|, $$

where ${\text{SD}}^{n} (g^{k} (t))$ and ${\text{SD}}^{n + 1} (g^{k} (t))$ denote the standard deviation of the gene expression for the center gene ${\text{g}}^{k}$ based on *n* reference samples and *n* + 1 mixed samples at time point *t*, respectively.

[Step 5] The fifth step is to calculate the global differential entropy $\Delta E(t)$, i.e.,

6 $$ \Delta E(t) = \frac{1}{Q}\sum\limits_{k = 1}^{Q} \Delta E^{k} (t)\Delta SD\left( {g^{k} (t)} \right), $$

where constant $Q$ denotes the number of all genes.$\Delta E(t)$ is called the global LNE score or LNE score since it can reflect the overall effect of a single sample. According to DNB theory [1, 2], when the system is close to the tipping point, the LNE score can effectively represent the fluctuations of the network and thus serve as an early warning sign of critical deterioration. Theoretical explanation of local network entropy (LNE) has been supplemented in the page S6 of Additional file 1.

### Data processing and functional analysis

Ten unrelated, clinical tumor datasets (kidney renal clear cell carcinoma (KIRC), lung squamous cell carcinoma (LUSC), stomach adenocarcinoma (STAD), liver hepatocellular carcinoma (LIHC), lung adenocarcinoma (LUAD), esophageal carcinoma (ESCA), colon adenocarcinoma (COAD), rectum adenocarcinoma (READ), thyroid carcinoma (THCA), and kidney renal papillary cell carcinoma (KIRP)) were downloaded from The Cancer Genome Atlas (TCGA) database (GDC) (cancer.gov https://portal.gdc.cancer.gov/). These datasets included RNA-seq data from tumor and tumor-adjacent samples and clinical information. The tumor samples were categorized into different stages according to the clinical (stage) information, samples lacking stage information were discarded, and the complete clinical staging information were provided in the Additional file 2: Table S1.

The molecular global template network was built with the following steps. First, the protein–protein interaction networks for Homo sapiens were downloaded from STRING (http://string-db.org). Second, the genes from each microarray dataset were mapped to the integrated network to construct the molecular network for subsequent analysis. Finally, the subsequent analysis results were visualized with Cytoscape (www.cytoscape.org).

The enrichment analysis was performed based on the KEGG Mapper tool (KEGG Mapper Color) and the DAVID Functional Annotation Tool (DAVID: Functional Annotation Tools (ncifcrf.gov). All statistical analyses were performed with R software v4.0.3 (R: The R Project for Statistical Computing (r-project.org)). The differential expression and Cox survival analyses were performed with the R DESeq2 package and R Survival package.

## Results

### Identifying the critical transition points of ten cancers with LNE

We used the LNE algorithm to identify the critical transition points of ten cancers in datasets that were downloaded from TCGA. The samples were categorized into different stages according to clinical information, and the tumor adjacent (TA) samples were used as reference samples for each tumor. For example, the KIRC samples were categorized into four stages, i.e., stages I, II, III, and IV. The LUSC samples were categorized into seven stages, i.e., stages IA, IB, IIA, IIB, IIIA, IIIB, and IV. The LNE score was then calculated for each single sample following the proposed algorithm, and the average LNE score curves of each stage are shown in Fig. 2A–J. Based on LNE theory, the transition point in the LNE score curve is the critical state during disease progression (Fig. 1A). To validate the identified critical state, the prognoses of before-transition and after-transition samples were determined and compared through Kaplan–Meier (log-rank) survival analysis (Fig. 2K–T). Regardless of specific biological and pathological differences, the stage II-III is a special state correlated with the lymph node metastasis, while the stage III-IV stands for a period associated with distant metastasis [19]. Therefore, we consider that stage II and stage III represent the critical point before lymph node metastasis and the critical stage before distant metastasis, respectively.

For KIRC, there was a drastic increase in the LNE score before stage III (transition point; Fig. 2A), suggesting the upcoming abrupt critical transition into the disease state (stage IV); that is, the stage at which the tumor migrates to form distant metastasis at stage III [20]. Furthermore, survival analysis was applied to compare survival curves for samples before and after the transition point (stage III) by log-rank tests to validate the identified critical state. The survival curves showed a significant difference (*p* < 0.0001) between stage I ~ III and stage IV KIRC samples according to clinical information (Fig. 2K). In addition, the survival time of patients after stage III was significantly shorter than that before stage III.

For LUSC, the average LNE score abruptly increased at stage IIB (Fig. 2B), indicating an upcoming critical transition after stage IIB; that is, after stage IIB, lymph node metastasis and tumor invasion of the visceral pericardial surface would occur [21]. Moreover, the survival curves of samples taken before and after the transition point (stage IIB) were significantly different (*p* = 0.0063; Fig. 2L). Furthermore, patients with diseases in stages after the identified critical transition had shorter survival times than those with diseases in stages before the transition.

For STAD, the average LNE score abruptly increased at stage IIIA (Fig. 2C), indicating an upcoming critical transition after stage IIIA; that is, the cancer would spread to the serosal layer of the stomach wall (stage IIIB) and ultimately cause distant metastasis (stage IV) [22]. Moreover, the survival curves of samples taken before and after the transition point (Stage IIIA) were significantly different (*p* = 0.0036; Fig. 2M). Furthermore, patients with diseases in stages after the identified critical state had shorter survival times than those with diseases in stages before the transition.

For LIHC, the average LNE score abruptly increased at stage II (Fig. 2D), suggesting an upcoming critical transition after stage II. A literature search showed that direct invasion of adjacent organs occurs at stage III [23]. Moreover, the survival curves of samples taken before and after the transition point (Stage II) were significantly different (*p* < 0.0001; Fig. 2N). Furthermore, patients with diseases in stages after the identified critical state had shorter survival times than those with diseases in stages before the transition.

The results of the same above-mentioned method for the other six cancers are shown in Fig. 2. Moreover, a method (Additional file 1: Fig. S5) was applied to validate the identified critical state, and the results were provided in the page S12 of Additional file 1. As demonstrated in these graphs, LNE is an effective method for identifying the critical state of cancers.

### The dynamic evolution of gene regulatory networks

Above, we use the numerical algorithm LNE to identify the critical state. Subsequently, we wanted to validate the identified critical states at the network level. For each sample, the top 5% of genes with the highest LNE score were chosen as the LNE genes. The common LNE genes of samples in the identified critical state were regarded as DNBs for consequent functional and biological analyses and may participate in key associations among biomolecules related to tumor deterioration during disease progression. First, these common LNE genes were mapped to the PPI network to further study the dynamic evolution of the network and to identify changes in cancer-related molecules.

**Fig. 3:**
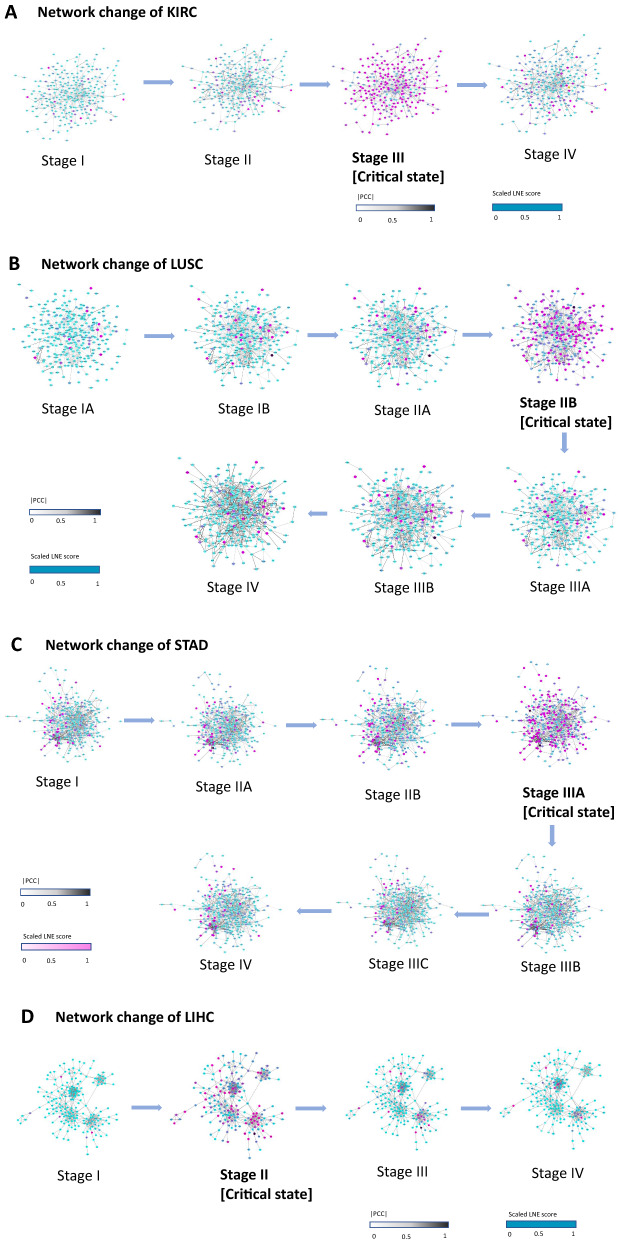
The dynamic network change of LNE genes in KIRC, LUSC, STAD and LIHC. **A** In KIRC, the LNE gene group/module evolved, and there was a significant change in the network structure at stage III. **B** Similarly, the network LNE score significantly changed at stage IIA in LUSC. **C** The network LNE score significantly changed at stage IIIA in STAD. **D** The network LNE score significantly changed at stage II in LIHC. The network structure was derived by mapping LNE genes to the STRING PPI network. We discarded all the isolated nodes without any links to other nodes. The color of each node denotes the value of the scaled local LNE score, while the color of each edge denotes the absolute value of the Pearson correlation coefficient $|{\text{PCC}}|$

For KIRC, the dynamic changes of gene’s LNE score in its PPI regulation network across all 4 stages are shown in Fig. 3A. An obvious change in the network structure occurred at stage III, indicating an upcoming critical transition, in line with the experimental results above. Furthermore, previous studies fully support that the critical state was correctly identified.

For LUSC, the dynamic changes of gene’s LNE score in the PPI regulation network across all 7 stages are shown in Fig. 3B. A drastic change in the PPI network occurred in stage IIB, indicating an upcoming critical transition, in line with the experimental results above.

For STAD, the dynamic changes of gene’s LNE score in the PPI regulation network across all 7 stages are shown in Fig. 3C. A drastic change in the PPI network occurred in stage IIIA, indicating an upcoming critical transition, in line with the experimental results above.

For LIHC, the dynamic changes of gene’s LNE score in its PPI regulation network across all 4 stages are shown in Fig. 3D. A drastic change in the PPI network occurred in stage II, indicating an upcoming critical transition, in line with the experimental results above.

The results in Fig. 3 also reflects some additional information in the network point of view. First, the evolution of the LNE gene group (*i.e.*, the top 5% genes with the highest LNE values) can signal the critical transition at the network level, that is, for the subnetwork composed of the LNE genes, a significant change in its topological structure occurs when the system approaches the critical state. Second, the LNE genes composed a connected subgraph when they were mapped to the protein–protein interaction (PPI) network, which can’t be implied by neither the LNE definition nor its mean value. Third, the changes of the network structure across all stages provide a clue of the dynamical evolution of the gene network during the disease progression, which may help to understand the underlying mechanisms of the associative relationships among the LNE genes.

Furthermore, results of the dynamic network changes for the other six cancers are presented in Additional file 1: Fig. S2, and also in line with the experimental results above.

### Prognostic prediction of tumors using LNE

LNE genes can be divided into two types of biomarkers: optimistic LNE (O-LNE) biomarkers and pessimistic LNE (P-LNE) biomarkers. Specifically, from a statistical perspective, those samples identified as O-LNE biomarkers tend to have an association with good prognosis, while those identified as P-LNE biomarkers tend to have an association with poor prognosis. More details about the identification method are provided in Fig. S1 and the page S1 of Additional file 1.

The predicted prognoses of samples with optimistic LNE biomarkers in their LNE genes were more favorable than those of other samples; that is, the survival time of these samples was expected to be longer than that of other samples. In contrast, the predicted prognoses of samples with pessimistic LNE biomarkers in their LNE genes were less favorable than those of other samples; that is, the survival time of these samples was expected to be shorter than that of other samples.

**Fig. 4:**
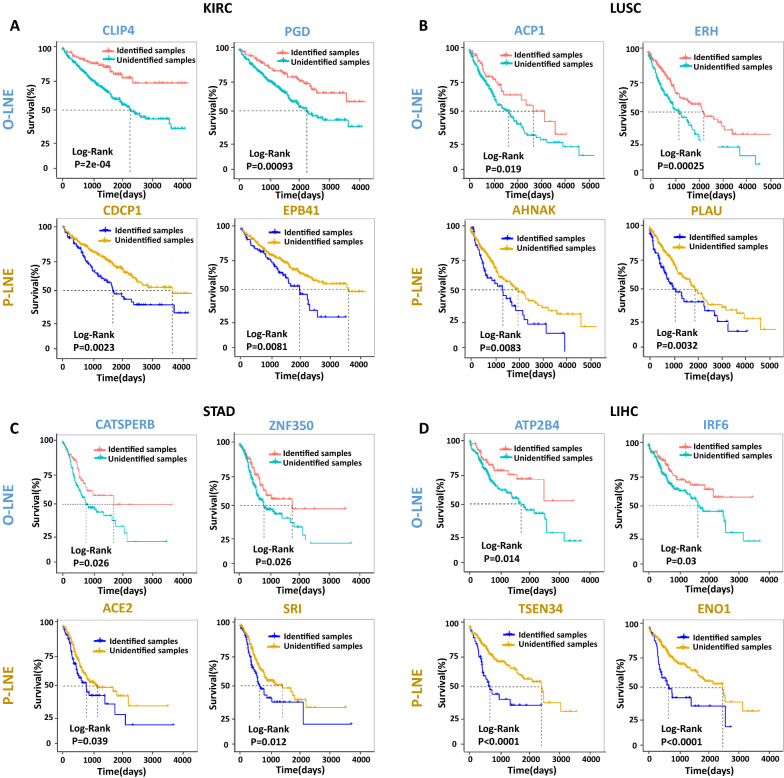
Comparison of survival curves between samples with and without O-LNE and P-LNE biomarkers for KIRC, LUSC, STAD, and LIHC. **A** The O-LNE biomarkers *CLIP4* and *PGD* and the P-LNE biomarkers *CDCP1* and *EPB41* in KIRC; **B** the O-LNE biomarkers *ACP1* and *ERH* and the P-LNE biomarkers *AHNAK* and *PLAU* in LUSC; **C** the O-LNE biomarkers *CATSPERB* and *ZNF350* and the P-LNE biomarkers *ACE2* and *SRI* in STAD; **D** the O-LNE biomarkers *ATP2B4* and *IRF6* and the P-LNE biomarkers *TSEN34* and *ENO1* in LIHC. Samples with O-LNE or P-LNE biomarkers in their LNE genes were deemed as samples with biomarkers, while other samples were deemed samples without biomarkers

For KIRC, the survival times of samples with the O-LNE biomarkers *CLIP4* (*p* = 0.0002; Fig. 4A) and *PGD* (*p* = 0.00093; Fig. 4A) were significantly longer than those of samples without these O-LNE biomarkers according to survival curve analysis (Fig. 4). The survival times of samples with the P-LNE biomarkers *CDCP1* (*p* = 0.0023; Fig. 4A) and *EPB41* (*p* = 0.0081; Fig. 4A) were significantly shorter than those of samples without these P-LNE biomarkers.

For LUSC, the prognoses of samples with the O-LNE biomarkers *ACP1* (*p* = 0.019; Fig. 4B) and *ERH* (*p* = 0.00025; Fig. 4B) tended to be significantly better than those of other samples; i.e., the survival time was expected to be longer. The prognoses of samples with the P-LNE biomarkers *AHNAK* (*p* = 0.0083; Fig. 4B) and *PLAU* (*p* = 0.011; Fig. 4B) tended to be significantly worse than those of other samples; i.e., the survival time was expected to be longer.

For STAD, those samples with the O-LNE biomarkers *CATSPERB* (*p* = 0.026; Fig. 4C) and *ZNF350* (*p* = 0.026; Fig. 4C) tended to have significantly better prognoses than other samples. Those samples with the P-LNE biomarkers *ACE2* (*p* = 0.039; Fig. 4C) and *SRI* (*p* = 0.012; Fig. 4C) tended to have significantly worse prognoses than other samples.

For LIHC, those samples with the O-LNE biomarkers *ATP2B4* (*p* = 0.014; Fig. 4D) and *IRF6* (*p* = 0.03; Fig. 4D) tended to have a significantly more optimistic prognosis than other samples. Those samples with the P-LNE biomarkers *TSEN34* (*p* < 0.0001; Fig. 4D) and *ENO1* (*p* < 0.0001; Fig. 4D) tended to have a significantly worse prognosis than other samples.

Furthermore, these novel biomarkers may play significant roles in essential biological processes during critical deterioration. As shown in Table 1, in KIRC, *CLIP4* was identified as an O-LNE biomarker; *CLIP4* is involved in regulating the expression of several tumor-associated genes, and its expression is considered to stimulate tumor metastasis [13]. In LUSC, *FGF11* was identified as an O-LNE biomarker; and *FGF11* may be involved in the stabilization of capillary-like tube structures associated with angiogenesis and may act as a modulator of hypoxia-induced pathological processes such as tumorigenesis [14]. In STAD, *ACE2* was identified as a P-LNE biomarker, and *ACE2* can affect macrophage expression of tumor necrosis factor (TNF-α) [15]. In LIHC, *TTK* was identified as a P-LNE biomarker, and *TTK* alone or in combination with other therapeutics may selectively kill tumor cells [16].

**Table 1:** Optimistic LNE (O-LNE) biomarkers and pessimistic LNE (P-LNE) biomarkers in KIRC, LUSC, STAD and LIHC

Cancer	Gene	Type	Family	Relation with cancer progression
KIRC	*CLIP4*	O-LNE	Regulatory	*CLIP4* is involved in regulating the expression of several tumor-associated genes, and its expression is considered to stimulate tumor metastasis [13]
	*CDCP1*	P-LNE	Enzyme	*CDCP1* belongs to the tetraspanin web involved in tumor progression and metastasis [24]
LUSC	*FGF11*	O-LNE	Signaling growth factor	*FGF11* may be involved in the stabilization of capillary-like tube structures associated with angiogenesis and may act as a modulator of hypoxia-induced pathological processes such as tumorigenesis [14]
	*AHNAK*	P-LNE	Regulatory	*AHNAK* mediates negative regulation of cell growth and acts as novel tumor suppressor through potentiation of TGFB1 signaling [25]
STAD	*ZNF350*	O-LNE	Transcription factor	*ZNF350* has been reported to function as a tumor suppressor [26]
	*ACE2*	P-LNE	Enzyme	*ACE2* can affect macrophage expression of tumor necrosis factor (TNF-α) [15]
LIHC	*IRF6*	O-LNE	Transcription factor	*IRF6* suppresses tumorigenesis in stratified epithelia [27]
	*TTK*	P-LNE	Enzyme	*TTK* alone or in combination with other therapeutics may selectively kill tumor cells [16]

The O-LNE and P-LNE biomarkers of the ten cancers are provided in Additional file 3: Table S2. The results imply that O-LNE and P-LNE biomarkers are effective in determining patient prognosis from a statistical perspective and may participate in essential biological processes. Moreover, these biomarkers are mainly non-differentially expressed genes and are therefore usually missed by traditional studies. For example, only *KIF2C* and *MYBL2* were differentially expressed genes in LIHC (Additional file 3: Table S2), and *GRB7* was differentially expressed in THCA (Additional file 3: Table S2). Hence, we present a method to identify novel biomarkers, drug targets and key regulators.

### Functional analysis of LNE genes in different systems

For cancers affecting the same system of the human body, common LNE genes identified in the critical state may participate in the same biological processes during tumor deterioration. In this study, the cancers were categorized into 3 groups based on the system affected, i.e., the respiratory system (LUSC and LUAD), urinary system (KIRP and KIRC), and digestive system (STAD, READ, LIHC, ESCA, and COAD).

**Fig. 5:**
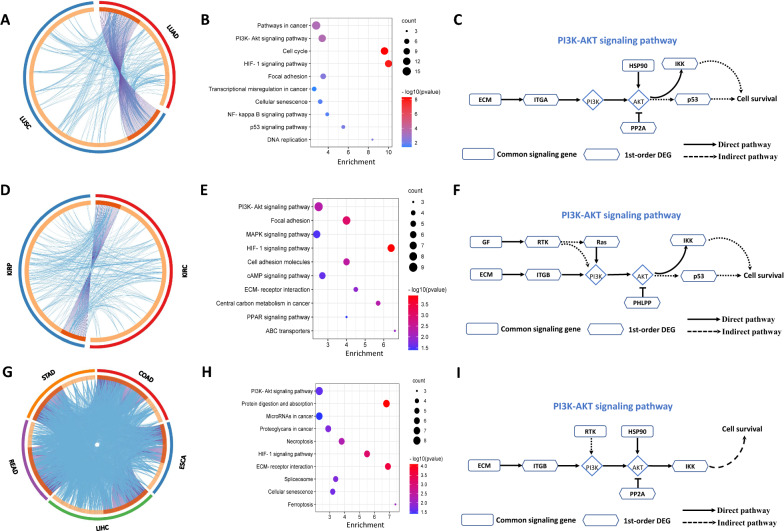
Common LNE genes in cancers affecting the same system of the human body in the critical state are involved in essential biological processes related to cancer. KEGG pathway enrichment analysis of common LNE genes (in the critical state) of cancers affecting different systems: **A** respiratory system; **D** urinary system; **G** digestive system. Determination of overlapping genes of from cancers affecting the same system and their functional assessment: **B** respiratory system; **E** urinary system; **H** digestive system. The outer ring denotes different cancers, and the inner ring denotes their identity and function. The identities are linked with each other with purple lines, and the functions are shown in blue. Regulatory patterns of the PI3K-Akt signaling pathway in the 3 body systems: **C** respiratory system; **F** urinary system; **I** digestive system

For each of the 3 systems, there were not only many overlapping the signaling genes in different cancers but also tight functional relationships among them (Fig. 5A, 5D, and 5G). For each system described above, KEGG pathway enrichment analysis of the common signaling genes among the different cancers was performed. For the 3 systems of the human body, as presented in Fig. 5B, 5E, and 5H, the common signaling genes were significantly enriched in pathways related to the process of cancer development. For the respiratory system (LUSC and LUAD), the common LNE genes were enriched in cancer-related pathways, including the *PI3K-Akt* signaling pathway, pathways in cancer, and the cell cycle pathway (Fig. 5B). Activation of the *PI3K-Akt* pathway and pathways in cancer has been reported to be involved in the development and progression of lung cancer [28]. In melanocytes driven by lung cancer cell-derived exosomes, the cell cycle pathway may contribute to tumor progression more than any other pathway [29]. For the urinary system (KIRP and KIRC) (Fig. 5E), the common LNE genes were enriched in pathways associated with cancer, such as the *PI3K-Akt* signaling pathway, focal adhesion pathway, and *MAPK* signaling pathway. Suppressing the *PI3K-Akt* signaling pathway can inhibit cell proliferation and induce apoptosis [30]. Activating the *MAPK* signaling pathway can promote the proliferation and invasion of renal cell carcinoma cells [31]. For the digestive system (STAD, READ, LIHC, ESCA, and COAD), the common LNE genes were enriched in cancer-related pathways, including the *PI3K-Akt* signaling pathway, microRNAs in cancer, and proteoglycans in cancer (Fig. 5H). The *PI3K-Akt* signaling pathway has been shown to drive tumor progression and regulate metastasis in multiple cancer cells [32, 33]. MicroRNAs are critical factors in cancer biology [34]. Proteoglycans can perform multiple functions in cancer and normal angiogenesis and are directly related to cancer [35, 36]. Although unique pathways were present among these 3 different systems, some common pathways were present as well, such as the *PI3K-Akt* signaling pathway. Moreover, we found that the regulatory patterns of the *PI3K-Akt* signaling pathway for these 3 systems were quite similar. Specifically, upstream regulators, such as factors in the extracellular matrix (*ECM*) (common signaling genes), activate the downstream factors *ITGA*/*ITGB* (1st-order DEGs), which subsequently activate the key downstream molecules *PI3KA* and *AKT* together with other common signaling genes and 1st-order neighboring DEGs; this cascade may trigger tumor development (Fig. 5C, F and I). In general, the synergy of common signaling genes and their 1st-order neighboring DEGs may have biological significance in tumor-related biological processes.

## Discussion

In biological studies and clinical practice, small sample sizes are an overarching problem, especially in cancer, and often contribute to model errors and bias in analyses. To avoid these problems when identifying transition points or critical states that appear just before the disease state, the local network entropy (LNE) method was applied in this study. Its effectiveness and robustness have been proven in various diseases.

We identified the tipping point of ten cancers from TCGA datasets by LNE. Survival analysis and analysis of dynamic changes in the mapped STRING network of LNE genes in the critical state confirm that the LNE method is viable, and LNE genes may participate in some key biological processes related to cancer. Through functional analysis and literature review, the common LNE genes in the critical state were found to be enriched in pathways related to tumor progression, such as the *ECM-*receptor interaction pathway, *PI3K-Akt* signaling pathway, and *HIF*-1 signaling pathway (Fig. 5).

In further analysis, we found that the LNE genes could be categorized into two novel types of biomarkers [37], i.e., pessimistic LNE (P-LNE) and optimistic LNE (O-LNE) biomarkers. We identified P-LNE and O-LNE biomarkers of the ten cancers, and these biomarkers could be used to evaluate patient prognosis. Accordingly, if a patient’s LNE genes included P-LNE biomarkers, the patient was likely to have shorter survival times; conversely, if a patient’s LNE genes included O-LNE biomarkers, the patient was likely to have longer survival times (Fig. 4). Furthermore, most of the O-LNE and P-LNE biomarkers that were identified lacked differential expression and would thus normally be missed by traditional analyses. For instance, for LIHC, *CYP2A6* and *INSIG1*, identified as O-LNE biomarkers, and *CKS2*, *ENO1*, *G6PD*, *TKT*, and *TSEN34*, identified as P-LNE biomarkers, all had nondifferential expression, and only *KIF2C* and *MYBL2*, identified as P-LNE biomarkers, showed differential expression. Through a literature search, these O-LNE and P-LNE biomarkers were found to be highly associated with biological processes related to cancer, such as promotion of tumor cell proliferation, migration, and formation. Thus, the P-LNE and O-LNE biomarkers identified here may be important targets for future research into the molecular mechanisms underlying tumor onset and/or disease state deterioration and will be topics of our future research. These findings of biomarker genes can be applied in many distinct biological questions of translation medicine, such as early detection of breast cancer [38], prediction of RNA-binding sites [39], network-based biomarker discovery [40], and detecting prognostic biomarkers of breast cancer [41].

There are several advantages of the LNE method. First, the proposed approach is model-free and does not need learning processes to identify biomarkers, which is different from traditional classification or machine learning methods, which require a large number of case/control samples for supervised or unsupervised learning. Second, our method can effectively identify the critical state of a complex disease at the single-sample level, benefiting the development of personalized medicine. Third, the LNE method provides practical biomarkers for prognostic analysis, which is helpful for finding new biomarkers, drug targets and prognostic indicators.

## Conclusions

The proposed computational method LNE effectively identified the critical transition state of complex diseases at the single-sample level, making it applicable for most real clinical data. Our method not only identified the critical state or transition point of ten cancers but also provides two types of new prognostic biomarkers, optimistic LNE (O-LNE) and pessimistic LNE (P-LNE) biomarkers, for further practical application. Hence, it has great potential in personalized diagnosis, the identification of the molecular mechanisms of disease progression, and prevention medicine.

## Supplementary Information

**Additional file 1.** Supplementary information of LNE.

**Additional file 2:** **Table S1.** The number of tumor samples within each stage in the cancer dataset from TCGA.

**Additional file 3:** **Table S2.** The optimistic LNE (O-LNE) biomarkers and pessimistic LNE (P-LNE) biomarkers in KIRC, LUSC, STAD, LIHC, LUAD, ESCA, COAD, READ, THCA, and KIRP.

## Data Availability

Kidney renal clear cell carcinoma (KIRC), lung squamous cell carcinoma (LUSC), stomach adenocarcinoma (STAD), liver hepatocellular carcinoma (LIHC), lung adenocarcinoma (LUAD), esophageal carcinoma (ESCA), colon adenocarcinoma (COAD), rectum adenocarcinoma (READ), thyroid carcinoma (THCA), and kidney renal papillary cell carcinoma (KIRP) are available from the cancer genome atlas (TCGA) database (http://cancergenome.nih.gov). The source code of algorithm is accessed in (https://github.com/liujuntan/LNE_Project).

## Abbreviations

LNE: Local network entropy
DNB: The dynamical network biomarker
LUAD: Lung adenocarcinoma
STAD: Stomach adenocarcinoma
THCA: Thyroid carcinoma
COAD: Colon adenocarcinoma
READ: Rectum adenocarcinoma
KIRC: Kidney renal clear cell carcinoma
LUSC: Squamous cell carcinoma
LIHC: Liver hepatocellular carcinoma
KIRP: Kidney renal papillary cell carcinoma
ESCA: Esophageal carcinoma
TCGA: The cancer genome atlas
SD: Standard deviation
PCC: Pearson correlation coefficient
LNE genes: The top 5% of genes with the highest LNE score
O-LNE biomarker: Optimistic LNE biomarker
P-LNE biomarker: Pessimistic LNE biomarker
