# Supplementary material for: Identifying the critical states and dynamic network biomarkers of cancers based on network entropy
Source: J Transl Med. 2022 Jun 6;20:254. doi: 10.1186/s12967-022-03445-0 (PMC9172070; doi:10.1186/s12967-022-03445-0)
Supplement: Supplementary file 1 — Additional file 1. Supplementary information of LNE. [file 12967_2022_3445_MOESM1_ESM.docx]

## Supplementary information of LNE

### Algorithm to identify the O-LNE/P-LNE biomarker

The schematic of the algorithm had been supplemented in Supplementary Figure S1 and can also be described as follow:

**[Step 1]** The first step is to categorize samples into a long-survival group and a short-survival group based on their clinical information, that is, the samples with a survival time greater than the median survival time were deemed as the long-survival group, and vice the samples with a survival time less than the median survival time were considered as the short-survival group.

**[Step 2]** The second step is to find candidate biomarkers. For each sample, the LNE genes (top 5% genes with the highest LNE value) were selected as a set of candidate genes. The samples were divided into two groups (identified samples and unidentified samples) according to whether their LNE genes contained the given candidate gene *i*, i.e., the LNE genes of identified samples included the given candidate gene *i,* while unidentified samples didn’t. The given candidate gene *i* is regarded as the candidate biomarker when it satisfies the following formula.

$\frac{n}{n+m}>A\text{ or }\frac{m}{n+m}>A$ (S1)

where *n* denotes the number of identified samples from the long-survival group, and *m* denotes the number of identified samples from the short-survival group. The A is an empirical parameter and set as 0.6.

**[Step 3]** The third step is to identify two types of biomarkers (optimistic LNE biomarkers and pessimistic LNE biomarkers) from candidate biomarkers. Specifically, for the candidate biomarker (gene *j*), the prognosis results respectively based on identified samples and unidentified samples were exhibited and compared through Kaplan-Meier (log-rank) survival analysis. If the survival curve of identified samples were significantly longer than that of unidentified samples (p-value <0.05), gene *j* was perceived as an optimistic LNE biomarker (O-LNE). Similarly, if the survival curve of identified samples was statistically shorter than that of unidentified samples (p-value <0.05), gene *j* was perceived as a pessimistic LNE biomarker (P-LNE).


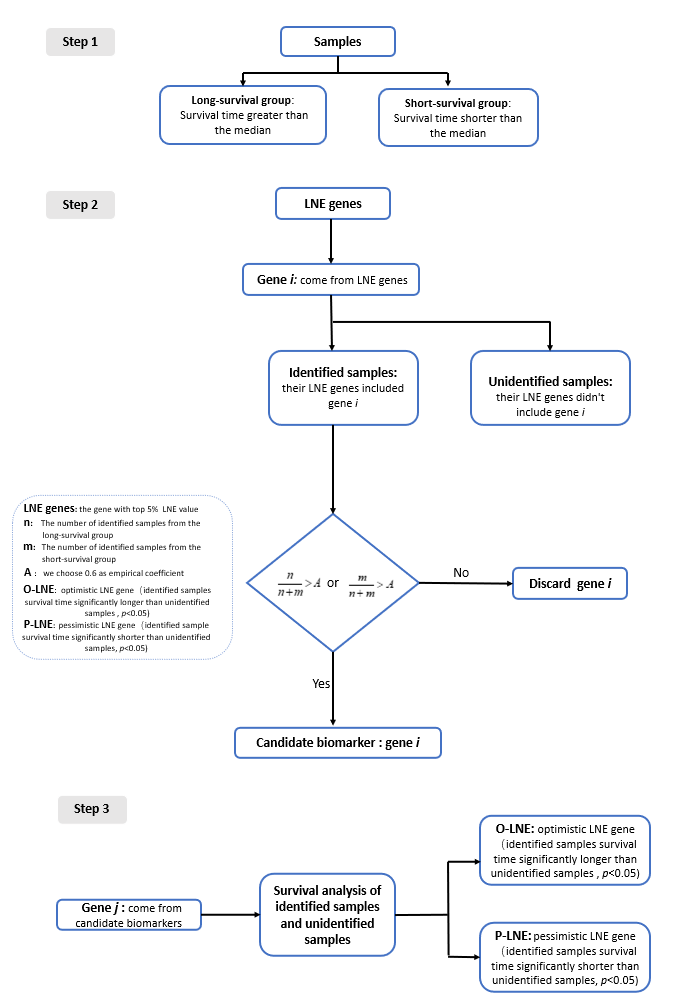


**Figure S1**: | **The schematic for identifying the O-LNE and P-LNE biomarkers.**

### The dynamic evolution of gene regulatory networks

Results of the dynamic network changes for the other six cancers. For KIRP, the dynamic changes of gene’s LNE score in its PPI regulation network across all 4 stages are shown in Fig. S2A. An obvious change in the network structure occurred at stage III, indicating an upcoming critical transition.


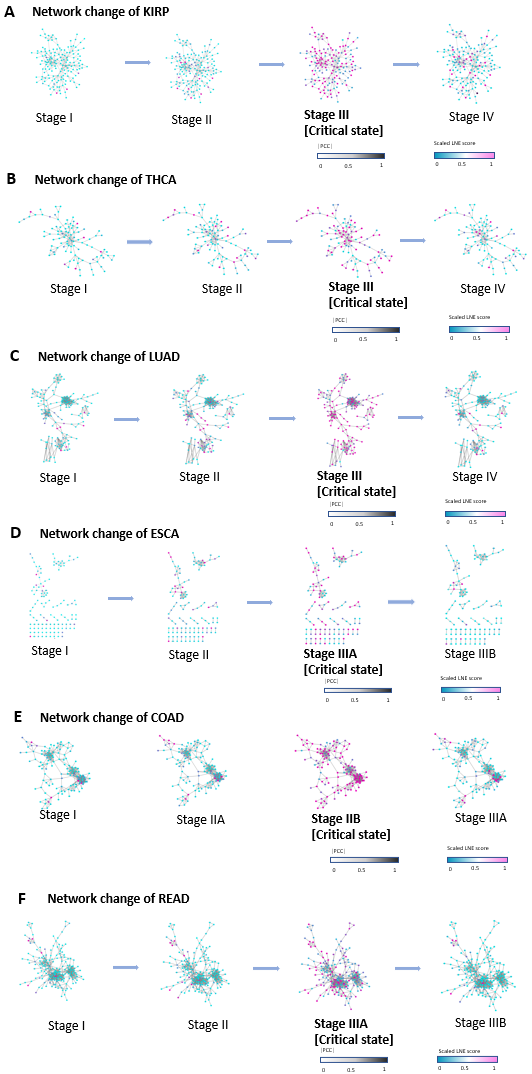


**Figure S2: | The dynamic network change of LNE genes in KIRP, THCA, LUAD, ESCA, COAD and READ.**

### O-LNE and P-LNE biomarkers of cancers

Accordingly, if a patient’s LNE genes included P-LNE biomarkers, the patient was likely to have shorter survival times; conversely, if a patient’s LNE genes included O-LNE biomarkers, the patient was likely to have longer survival times.


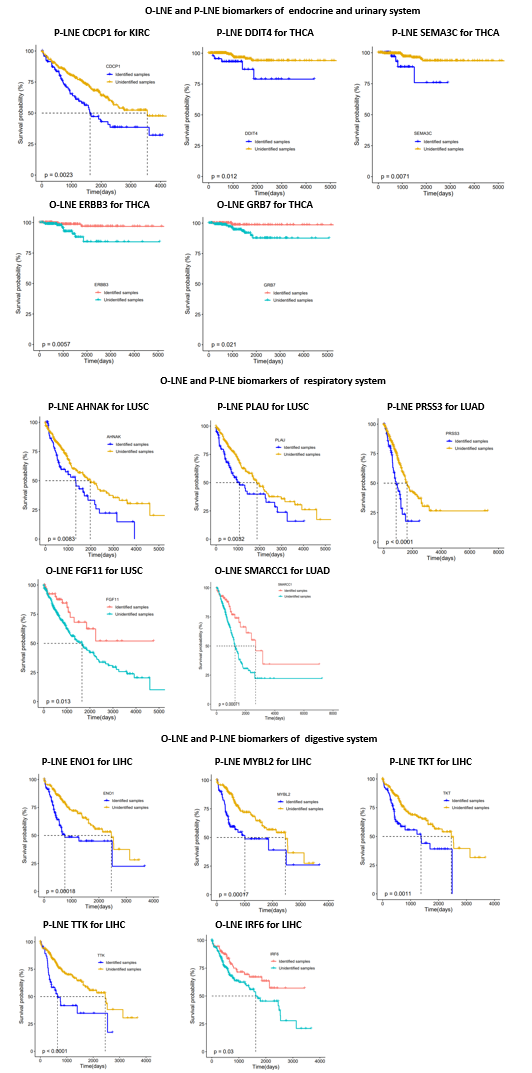


**Figure S3: | Comparison of survival curves between samples with and without O-LNE and P-LNE biomarkers for endocrine system, urinary system, respiratory system, and digestive system.**

### Revealing dark genes by LNE

New biomarkers, drug targets and key regulators are found by differential genes in most studies. Abnormally, the LNE methodology shows that some non-differential genes may also play essential roles in biological processes at the network level and should not be ignored. Therefore, we use survival analysis to detect these' dark genes' according to their LNE scores.

To begin with, we select LNE genes in critical state that are non-differential as alternative genes to reveal dark genes that are non-differentially at the expression level but sensitive to the LNE value. Based on R survival packages, we calculate the survival risk (Hazard ratio, HR). of each alternative gene by Cox model and log-rank test, those genes with p-value less than 0.05 were chosen. Samples were divided into high and low groups according to the median of chosen gene LNE score. The survival curves of the two groups were fitted and a log-rank test was made (p-value less than 0.05 were retained as alternative dark genes; Fig S4). In addition, survival analysis of these dark genes was also carried out in expression level, we divide samples into high and low expression groups according to the median of alternative dark gene expression, the survival curves of the two groups were fitted and log-rank test was made, we found these dark genes couldn’t distinguish the survival time between high and low group in expression level (Fig S4). In detail, as to KIRC, the survival time of the low LNE score group in APLP2 (p=0.038; Fig S4A) and BCAM (p=0.043; Fig S4A) significantly longer than the high SLE score group. In addition, the survival time between low expression group and high expression group didn't manifest significant difference in APLP2 (p=0.36; Fig S4A) and BCAM (p=0.69; Fig S4A). The resemble results of LUSC and STAD are shown in Fig S4. It's confidently approved that those dark genes have effectiveness in prognosis during disease progression in the LNE score level according to the results shown above.

These dark genes, sensitive to LNE score, may play important roles in essential biological processes during the critical deterioration. FERMT1 in LUSC, overexpressed in colon carcinoma cells may enhance tumor cell growth and matrix invasion (Kiriyama et al., 2013), promotes gastric cancer progression by activating the NF-κB pathway and predicts poor prognosis, suggest that FERMT1 might be a promising target for gastric cancer treatment (Fan et al., 2020). GAPDH and SLC2A1 in LUAD are enriched in HIF-1 signaling pathway and so on. But these "dark genes" usually be ignored since they are the non-differential genes. Hence, our approach provides the possibility of finding new biomarker, drug targets and prognosis indicators based on LNE score level.

Kiriyama, K. , Hirohashi, Y. , Torigoe, T. , Kubo, T. , & Sato, N. . (2013). Expression and function of fermt genes in colon carcinoma cells. Anticancer research, 33(1), 167-173.

Fan, H., Zhang, S., Zhang, Y., Liang, W., & Cao, B. (2020). FERMT1 promotes gastric cancer progression by activating the NF-κB pathway and predicts poor prognosis. *Cancer biology & therapy*, *21*(9), 815–825. <https://doi.org/10.1080/15384047.2020.1792218>


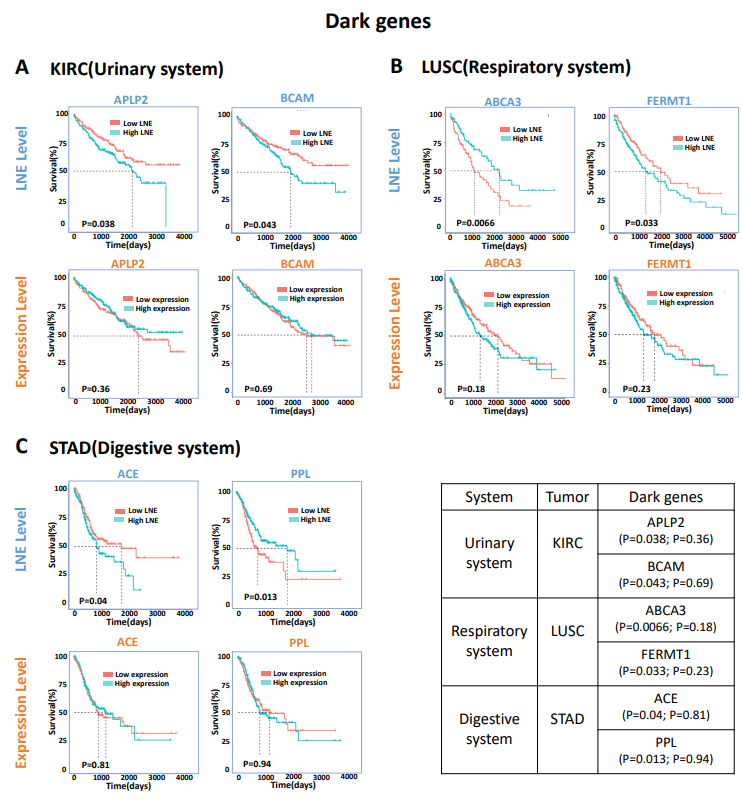


**Figure S4: | Dark genes for KIRC, LUSC, and STAD.**

### Theoretical explanation of local network entropy (LNE)

To theoretically explain how the local network entropy (LNE) works, denote $z_{i}$ as the expression of a biomolecule *i* (the expression value of a gene or a protein). According to DNB theory, when the biological system approaches a tipping point, the following results simultaneously hold:

1. For any biomolecule *i* in the DNB, the fluctuation of $z_{i}$ increases sharply, that is, the standard deviation

SD(*z_i_*) ↑; (S2)

1. If both biomolecules *i* and *j* are in the DNB, then the correlation between $z_{i}$ and $z_{j}$ sharply increases, that is, the Pearson correlation coefficient

|PCC(*z_i_,z_j_*)| ↑; (S3)

1. If *i* is in the DNB but *j* is not, then the correlation between $z_{i}$ and $z_{j}$ decreases sharply, that is, the Pearson correlation coefficient

|PCC(*z_i_,z_j_*)| ↓; (S4)

For two biomolecules *i* and *j* which both do not belong to the DNB, neither the standard deviations nor the correlation between $z_{i}$ and $z_{j}$ shows significant change.

Given a number of reference samples {s_1_(*t*), s_2_(*t*), …, s*_n_*(*t*)}, which are all generated from the normal state, far from the critical transition point. Note that if there are no such reference samples, then the LNE method is not applicable. According to the LNE algorithm presented in Methods section of the revised manuscript (i.e. the detailed five steps of calculating LNE), we focus on a local network N*^k^* which is centered at a gene $g^{k}$, whose 1st-order neighbors {$g_{1}^{k}$, $g_{2}^{k}$,…, $g_{M}^{k}$} are the *M* leaves.

If the single case sample s_case_(*t*) is in a normal state, i.e., a sampling point far from the critical state, then there is no significant change in the local LNE

$\Delta E(k,t)=\Delta\mathrm{SD}(g^{k}(t))|E^{n+1}(k,t)-E^{n}(k,t)|$, (S5)

with

$\Delta SD\left(g^{k}(t)\right)=\left|SD^{n}\left(g^{k}(t)\right)-SD^{n+1}\left(g^{k}(t)\right)\right|$, (S6)

where $\mathrm{SD}^{n}(g^{k}(t))$ and $\mathrm{SD}^{n+1}(g^{k}(t))$ are the standard deviations of center gene $g^{k}$ based on the *n* reference samples {s_1_(*t*), s_2_(*t*), …, s_n_(*t*)} and the *n*+1 samples {s_1_(*t*), s_2_(*t*), …, s_n_(*t*), s_case_(*t*)}, respectively. The differential entropy $|E^{n+1}(k,t)-E^{n}(k,t)|$ between $E^{n}(k,t)$ and $E^{n+1}(k,t)$ in Eq.(S5) characterizes the difference caused by the single sample s_case_(*t*), where

$E^{n+1}(k,t)=-\frac{1}{M}\sum_{i=1}^{M} p_{i}^{n+1}(t)\log p_{i}^{n+1}(t)$, (S7)

with

$p_{i}^{n\text{+}1}(t)=\frac{|\mathrm{PCC}^{n\text{+}1}(g_{i}^{k}(t),g^{k}(t))|}{\sum_{j=1}^{M} |\mathrm{PCC}^{n\text{+}1}(g_{j}^{k}(t),g^{k}(t))|}$, (S8)

and

$E^{n}(k,t)=-\frac{1}{M}\sum_{i=1}^{M} p_{i}^{n}(t)\log p_{i}^{n}(t)$. (S9)

with

$p_{i}^{n}(t)=\frac{|\mathrm{PCC}^{n}(g_{i}^{k}(t),g^{k}(t))|}{\sum_{j=1}^{M} |\mathrm{PCC}^{n}(g_{j}^{k}(t),g^{k}(t))|}$. (S10)

This is because that s_case_(*t*) and the reference samples {s_1_(*t*), s_2_(*t*), …, s*_n_*(*t*)} all belong to the normal state that is far from the critical point. At the normal state, the expression of a gene in either s_case_(*t*) or s*_l_*(*t*) (*l*=1,2,…,*n*) is a value approximately equal to its average value considering that the normal state is a stable equilibrium and the expression deviation of each gene is small.

However, when the single case sample s_case_(*t*) is in a pre-disease state, i.e., a sampling point near the tipping point just before the critical transition, there is a significant change in the local LNE if the nodes in the local network N*^k^* are all DNB members. Specifically, as showed in Eq. (S2), the drastically fluctuating expression of each DNB member leads to the increase of $\Delta\mathrm{SD}(k,t)$ in Eq. (S6), since the expression of each gene in the new coming sample s_case_(*t*) deviates largely from the average value with relatively high probability, which can be easily proven. On the other hand, as shown in Eq. (S3), the correlations between the DNB genes/members in the new coming sample s_case_(*t*) in terms of absolute value sharply increase, and thus $|\mathrm{PCC}^{n\text{+}1}(g_{i}^{k}(t),g^{k}(t))|$ increases more if $|\mathrm{PCC}^{n}(g_{i}^{k}(t),g^{k}(t))|$ is smaller among *M* edges (or $|\mathrm{PCC}^{n\text{+}1}(g_{i}^{k}(t),g^{k}(t))|$ increases less if $|\mathrm{PCC}^{n}(g_{i}^{k}(t),g^{k}(t))|$ is larger among *M* edges). Therefore, the differences (in absolute values) between the two correlation coefficients among the *M* edges for the *n*+1 samples become smaller, or all the correlation coefficients $|\mathrm{PCC}^{n\text{+}1}(g_{i}^{k}(t),g^{k}(t))|$ (*i*=1, 2, …, *M*) tend to be more similar comparing to $|\mathrm{PCC}^{n}(g_{i}^{k}(t),g^{k}(t))|$, which results in the increase of *E^n+1^*(*k,t*) and thus the increase of term $|E^{n+1}(k,t)-E^{n}(k,t)|$ in Eq. (S5).

Next we show, the smaller $|\mathrm{PCC}^{n}(g_{i}^{k}(t),g^{k}(t))|$ is, the more $|\mathrm{PCC}^{n\text{+}1}(g_{i}^{k}(t),g^{k}(t))|$ increases among the *M* edges, if the single case sample s_case_(*t*) is in a pre-disease state. Specifically, we denote $X=\{x_{1}, x_{2},\ldots, x_{n}\}$ and $Y=\{y_{1}, y_{2},\ldots, y_{n}\}$ as two genes in the local network N*^k^*. All of these *n* reference samples are assumed to be in a group with the same phenotype, attribute, or probability distribution. $\bar{X}_{n}\text{=}\frac{1}{n}\sum_{i\text{=}1}^{n} x_{i}$ is the mean of $X=\{x_{1}, x_{2},\ldots,x_{n}\}$, while $\bar{X}_{n+1}=\frac{1}{n+1}\sum_{i\text{=}1}^{n+1} x_{i}$ is the mean of $X=\{x_{1}, x_{2},\ldots, x_{n}, x_{n\text{+}1}\}$.

For the local network N*^k^*, there are *M* edges. According to Eq. (S8) and Eq. (S10), the probability for each edge is set as $p_{i}^{n}(t)=\frac{|R_{i}^{n}|}{\sum_{j=1}^{M} |R_{j}^{n}|}$ and $p_{i}^{n\text{+}1}(t)=\frac{|R_{i}^{n\text{+}1}|}{\sum_{j=1}^{M} |R_{j}^{n\text{+}1}|}$, *i*=1, 2,…, *M*, with

$R_{i}^{n}=\text{PCC}_{i}^{n}(X,Y)=\frac{\sum_{j\text{=}1}^{n} (x_{j}-\bar{X}_{n})(y_{j}-\bar{Y}_{n})}{\sqrt{\sum_{j=1}^{n} (x_{j}-\bar{X}_{n})^{2}}\sqrt{\sum_{i=1}^{n} (y_{j}-\bar{Y}_{n})^{2}}}$, *i*=1, 2,…, *M*, (S11)

and

$R_{i}^{n\text{+}1}=\text{PCC}_{i}^{n+1}(X,Y)=\frac{\sum_{j=1}^{n\text{+}1} (x_{j}-\bar{X}_{n\text{+}1})(y_{j}-\bar{Y}_{n\text{+}1})}{\sqrt{\sum_{j=1}^{n\text{+}1} (x_{j}-\bar{X}_{n\text{+}1})^{2}}\sqrt{\sum_{j=1}^{n\text{+}1} (y_{j}-\bar{Y}_{n\text{+}1})^{2}}}$, *i*=1, 2,…, *M*, (S12)

where the superscripts *n* and *n*+1 represent that the Pearson Correlation Coefficients (PCC) are based on the reference samples {s_1_(*t*), s_2_(*t*), …, s_n_(*t*)} and mixed samples {s_1_(*t*), s_2_(*t*), …, s_n_(*t*), s_case_(*t*)}, respectively. Clearly, the probabilities satisfy

$\sum_{i\text{=}1}^{M} p_{i}^{n}(t)=1$, $p_{i}^{n}(t)>0$, and $\sum_{i\text{=}1}^{M} p_{i}^{n\text{+}1}(t)=1$, $p_{i}^{n\text{+}1}(t)>0$. (S13)

When the system is at the critical state, there are the following cases for the local network N*^k^* from DNB property Eq. (S3):

- If the original correlation coefficient $|R_{i}^{n}|$ (from the reference samples $X^{n}=\{x_{1}, x_{2},\ldots, x_{n}\}$ and $Y^{n}=\{y_{1}, y_{2},\ldots, y_{n}\}$) is small, the difference |$| R_{i}^{n\text{+}1}| -|R_{i}^{n}|$| becomes large among *M* edges, due to the increase of the correlation (in absolute value) between *X* and *Y* for the additional sample *n*+1 at the critical state.
- If the original correlation coefficient $|R_{i}^{n}|$ (from the reference samples $X^{n}=\{x_{1}, x_{2},\ldots,x_{n}\}$ and $Y^{n}=\{y_{1}, y_{2},\ldots,y_{n}\}$) is large, the difference |$| R_{i}^{n\text{+}1}| -|R_{i}^{n}|$| becomes small among *M* edges.

We can approximately describe those cases in the following way:

$\text{|}R_{i}^{n\text{+}1}| -|R_{i}^{n}|=\frac{\Delta R}{\text{|}R_{i}^{n}|}$, (S14)

with $\Delta R>0$ when the system is in the critical state, that is, at the critical state the increment of correlation coefficient $|R_{i}^{n\text{+}1}| -|R_{i}^{n}|$is inversely proportional to $|R_{i}^{n}|$. Thus

$\begin{matrix} p_{i}^{n\text{+}1}\text{=} \frac{|R_{i}^{n\text{+}1}|}{\sum_{k=1}^{M} |R_{k}^{n\text{+}1}|}=\frac{|R_{i}^{n}|+\frac{\Delta R}{\text{|}R_{i}^{n}|}}{\sum_{k=1}^{M} \left[|R_{k}^{n}|+\frac{\Delta R}{\text{|}R_{k}^{n}|}\right]}=\frac{\frac{|R_{i}^{n}|}{\sum_{k=1}^{M} |R_{i}^{n}|}+\frac{\Delta R}{\text{|}R_{i}^{n}|}\frac{\sum_{k=1}^{M} |R_{i}^{n}|}{(\sum_{k=1}^{M} |R_{i}^{n}|)^{2}}}{1+\frac{\Delta R}{\sum_{k=1}^{M} |R_{i}^{n}|}\sum_{k=1}^{M} \left[\frac{\sum_{k=1}^{M} |R_{i}^{n}|}{|R_{k}^{n}|}\frac{1}{\sum_{k=1}^{M} |R_{i}^{n}|}\right]}\text{=}\frac{p_{i}^{n}+\frac{1}{p_{i}^{n}}\frac{\Delta R}{(\sum_{k=1}^{M} |R_{i}^{n}|)^{2}}}{1+\frac{\Delta R}{(\sum_{k=1}^{M} |R_{i}^{n}|)^{2}}\sum_{k=1}^{M} \left[\frac{1}{p_{i}^{n}}\right]} \\ \end{matrix}$ (S15)

Let $\Delta x=\frac{\Delta R}{(\sum_{k=1}^{M} |R_{i}^{n}|)^{2}}$, $a=\sum_{j=1}^{M} \frac{1}{p_{j}^{n}}$ and $Z_{n}=\{p_{1}^{n}, p_{2}^{n},\ldots,p_{M}^{n}\}$. Substituting $\frac{1}{1+a \Delta x}=1-a \Delta x+O(\Delta x^{2})$ into Eq. (S15), it follows

$p_{i}^{n\text{+}1}\text{=} \frac{p_{i}^{n}+\frac{\Delta x}{p_{i}^{n}}}{1+a \Delta x}=p_{i}^{n}+\frac{\Delta x}{p_{i}^{n}}-a p_{i}^{n}\Delta x+O(\Delta x^{2})$. (S16)

There is

$\begin{matrix} \text{SD}(Z_{n+1})^{2}-\text{SD}(Z_{n})^{2}=\frac{\sum_{j=1}^{M} \left(p_{j}^{n+1}-\bar{Z}_{n+1}\right)^{2}}{M}-\frac{\sum_{j=1}^{M} (p_{j}^{n}-\bar{Z}_{n})^{2}}{M} \\ =\frac{1}{M}\sum_{j=1}^{M} \left[\left(p_{j}^{n+1}-\frac{\sum_{k=1}^{M} p_{k}^{n+1}}{M}\right)^{2}-\left(p_{j}^{n}-\frac{\sum_{k=1}^{M} p_{k}^{n}}{M}\right)^{2}\right] \\ =\frac{1}{M}\sum_{j=1}^{M} \left[\left(p_{j}^{n+1}-\frac{1}{M}\right)^{2}-\left(p_{j}^{n}-\frac{1}{M}\right)^{2}\right] \\ \text{=}\frac{1}{M}\sum_{j=1}^{M} \left[\left(p_{i}^{n}+\frac{\Delta x}{p_{i}^{n}}-a p_{i}^{n}\Delta x+O(\Delta x^{2})-\frac{1}{M}\right)^{2}-\left(p_{j}^{n}-\frac{1}{M}\right)^{2}\right] \\ \text{=}\frac{1}{M}\sum_{j=1}^{M} \left[\left(\frac{\Delta x}{p_{i}^{n}}-a p_{i}^{n}\Delta x\right)\left(2p_{j}^{n}-\frac{2}{M}+\frac{\Delta x}{p_{i}^{n}}-a p_{i}^{n}\Delta x\right)+O(\Delta x^{2})\right] \\ \text{=}\frac{2\Delta x}{M}\sum_{j=1}^{M} \left[1-a (p_{j}^{n})^{2}+\frac{a p_{i}^{n}}{M}-\frac{1}{Mp_{i}^{n}}\right]+O(\Delta x^{2}) \\ \text{=}\frac{2\Delta x}{M}\sum_{j=1}^{M} \left[1-a (p_{j}^{n})^{2}\right]+\frac{2\Delta x}{M^{2}}\left[a \sum_{j=1}^{M} p_{i}^{n}-\sum_{j=1}^{M} \frac{1}{p_{i}^{n}}\right]+O(\Delta x^{2}) \\ \text{=}\frac{2\Delta x}{M}\sum_{j=1}^{M} \left[1-(p_{j}^{n})^{2}\sum_{j=1}^{M} \frac{1}{p_{i}^{n}} \right]+O(\Delta x^{2}) \end{matrix}$ (S17)

by reminding that $\sum_{j=1}^{M} p_{j}^{n}\text{=}1$ and $a=\sum_{j=1}^{M} \frac{1}{p_{j}^{n}}$.

For the following maximization problem

$\left\{\begin{matrix} \mathop{\text{Max}}\limits_{p_{1}^{n},p_{2}^{n},...,p_{M}^{n}} \sum_{j=1}^{M} \left[1-(p_{j}^{n})^{2}\sum_{k=1}^{M} \frac{1}{p_{k}^{n}}\right] \\ \text{s.t.} \sum_{j=1}^{M} p_{k}^{n}\text{=}1, p_{k}^{n}\geq0 \end{matrix}\right.$, (S18)

it is clear that when $p_{1}^{n}=p_{2}^{n}=\cdots=p_{M}^{n}=\frac{1}{M}$, there is maximum value 0 for Eq. (S18). Therefore, $\text{SD}(Z_{n+1})^{2}-\text{SD}(Z_{n})^{2}\leq0$, that is, when we add one sample (at the critical state) to the reference samples, the standard deviation of $Z_{n\text{+}1}\text{=}\{p_{1}^{n\text{+}1}, p_{2}^{n\text{+}1},\ldots, p_{M}^{n\text{+}1}\}$ is smaller than that of $Z_{n}=\{p_{1}^{n}, p_{2}^{n},\ldots, p_{M}^{n}\}$, which implies that the probabilities $p_{1}^{n\text{+}1}, p_{2}^{n\text{+}1},\ldots, p_{M}^{n\text{+}1}$ become more similar or equalized than $p_{1}^{n}, p_{2}^{n},\ldots, p_{M}^{n}$. Thus, $E^{n+1}(k,t)=-\frac{1}{M}\sum_{i=1}^{M} p_{i}^{n+1}(t)\log p_{i}^{n+1}(t)$ is larger than $E^{n}(k,t)=-\frac{1}{M}\sum_{i=1}^{M} p_{i}^{n}(t)\log p_{i}^{n}(t)$, which shows our conclusion.

When LNE is applied to a real dataset, it is unknown whether or not a local network contains DNB molecules. To detect the critical signal, we choose those local networks with the most significant changes in local LNE values.

### Method to validate the identified critical state

We applied a schematic method to validate the identified critical state. Specifically, if the identified critical state by LNE is IIA, then survival test 1 is to compare those before-transition period (IA-IIA) samples with after-transition period (IIIB-IV) samples (the results were supplemented in Fig 2), survival test 2 is to compare any two stages in before-transition, survival test 3 is to compare any two stages in after-transition, and survival test 4 is to compare the critical point (IIA) with the next stage of the critical point (IIB). Furthermore, when survival test 1 and survival test 4 have a significant difference in survival time, that means the identified critical state is highly related to survival analysis, when survival test 2 and survival test 3 have little significant difference in survival time, that means there is no other stage related to critical transition in survival time. The schematic method was supplemented in Supplementary Figure S5. The schematic method was applied to KIRC, LIHC, KIRP, LUSC and ESCA, the results were provided in Supplementary Figure S6.

For LUSC, the critical state identified by LNE was stage IIB. The survival time of samples from the before-transition stage (stages I-IIB) and that of after-transition samples (samples from stages IIIA-IV) have a significant difference (P=0.0063; Fig. S6A). Furthermore, patients with diseases in stages after the identified critical transition had shorter survival times than those with diseases in the before-transition stage. For the samples solely from the two stages around the critical transition point, *i.e.*, stages IIB and IIIA, the survival time of stage-IIB samples is longer than that of stage-IIIA samples (P=0.051; Fig. S6B). Besides, to check if there is any other critical transition that leads to different survival time, a series of survival analysis has been carried out. Specifically, the survival curves of samples from before-transition stages (stage I and stage IA) have little significant difference (P=0.92; Fig. S6C), and there were no significant differences (P=0.16; Fig. S6D) in survival curves among samples in stages IIIA, IIIB, and IV (the after-transition stage).

For ESCA, stage IIIA was identified as the critical state by LNE. As shown in Fig. S6E, there is a significant difference (*P*<0.0001) between the survival curves of samples before and after stage IIIA of ESCA. Clearly, the survival time of before-transition samples (samples from stages I-IIIA) is considerably longer than that of after-transition samples (samples from stages IIIB-IV). Furthermore, there has a significant difference (*P*=0.032; Fig. S6F) in the survival curves between the critical point (stage IIIA) and its next stage (IIIB). However, the survival curves of samples from before-transition stages (stage I and stage II) have little significant difference (*P*=0.24; Fig. S6G), and the survival curves of samples from the after-transition stages (stage IIIB and stage IV) have little significant difference (*P*=0.087; Fig. S6H), which confirmed there is no other critical transition that leads to different survival time.

For KIRC, the identified critical state by LNE was stage III. The survival tests were applied to validate the critical state. On the one hand, the survival time of before-transition samples (samples from stages I-III) and that of after-transition samples (samples from stages IV) have a significant difference (P<0.0001; Fig. S6I), and the survival curve between the critical point (stage III) and its next stage (IV) has a significant difference (P<0.0001; Fig. R3J). On the other hand, the survival curves of samples from before-transition stages (stage I and stage II) have little significant difference (P=0.59; Fig. S6K). Taken together, which confirm stage III was the critical transition that results in different survival time and there is no other critical transition.

For LIHC, stage II was identified as the critical state by LNE. Fig. S6L shows that there is a significant difference (P<0.0001) between the survival time of two groups of samples, *i.e.*, samples respectively from the before-transition period (stages I-II) and the after-transition period (stages III-IV) of LIHC. Moreover, it is also noted that the survival time of samples from the critical point (stage II) has a significant difference (P=0.046; Fig. S6M) than that of samples from stage III. However, the survival time of samples from after-transition stages (stage III and stage IV) have little significant difference (P=0.14; Fig. S6N). These results demonstrated that stage II was the critical transition of LIHC.

For KIRP, the identified critical state by LNE was stage III. Specifically, the survival time of before-transition samples (samples from stages I-III) and that of after-transition samples (samples from stages IV) have a significant difference (*P*<0.0001; Fig. S6O). In addition, the survival time of samples from critical point (stage III) has a significant difference (*P*=0.00097; Fig. S6P) than that of samples from stage IV. However, the survival time of samples from before-transition (stage I and stage II) have little significant difference (*P*=0.62; Fig. S6Q). These results demonstrated that stage III was the early warning signal associated with disease deterioration in KIRP.


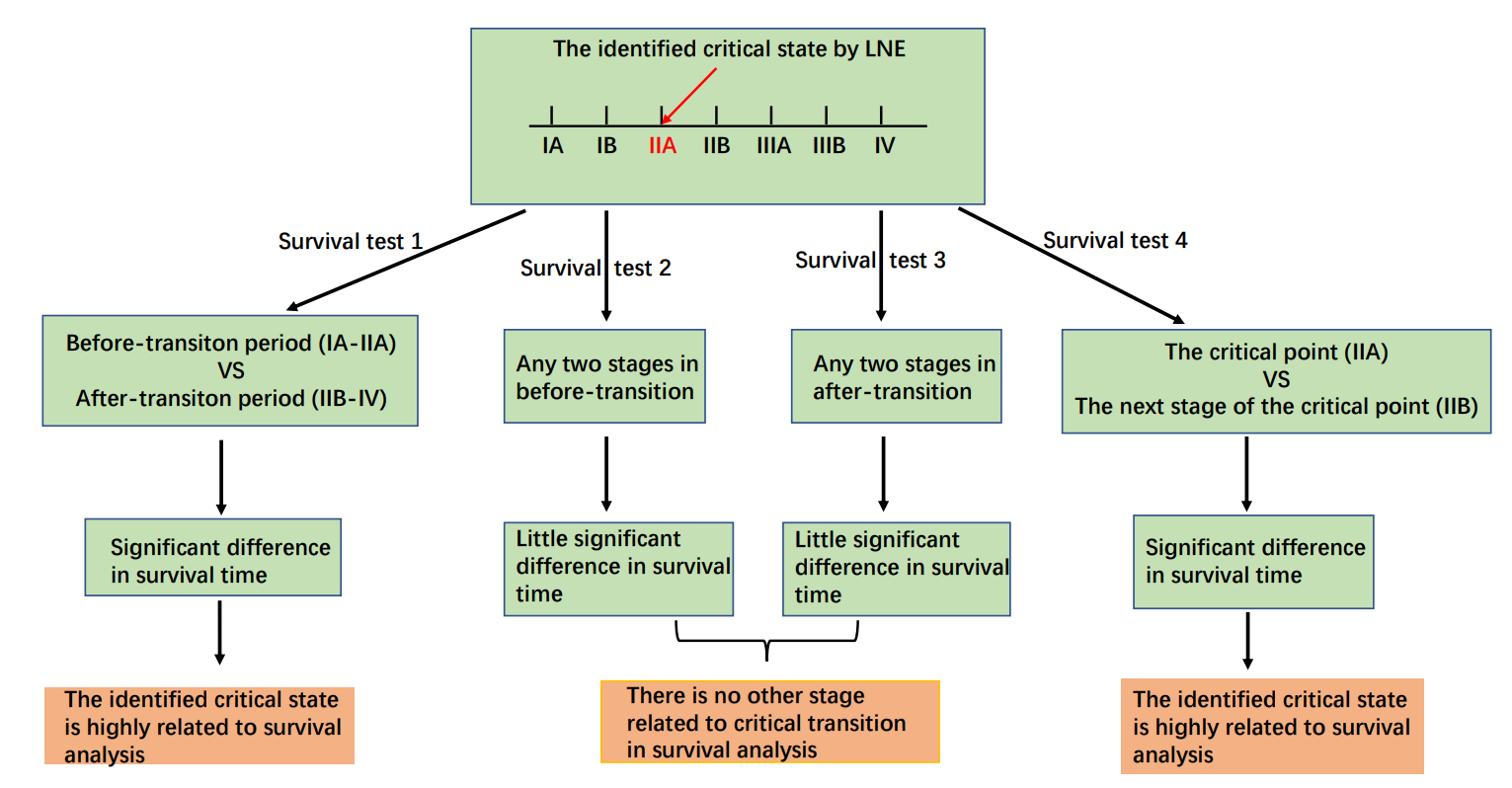


**Figure S5: |** **The schematic for validating the identified critical state.**


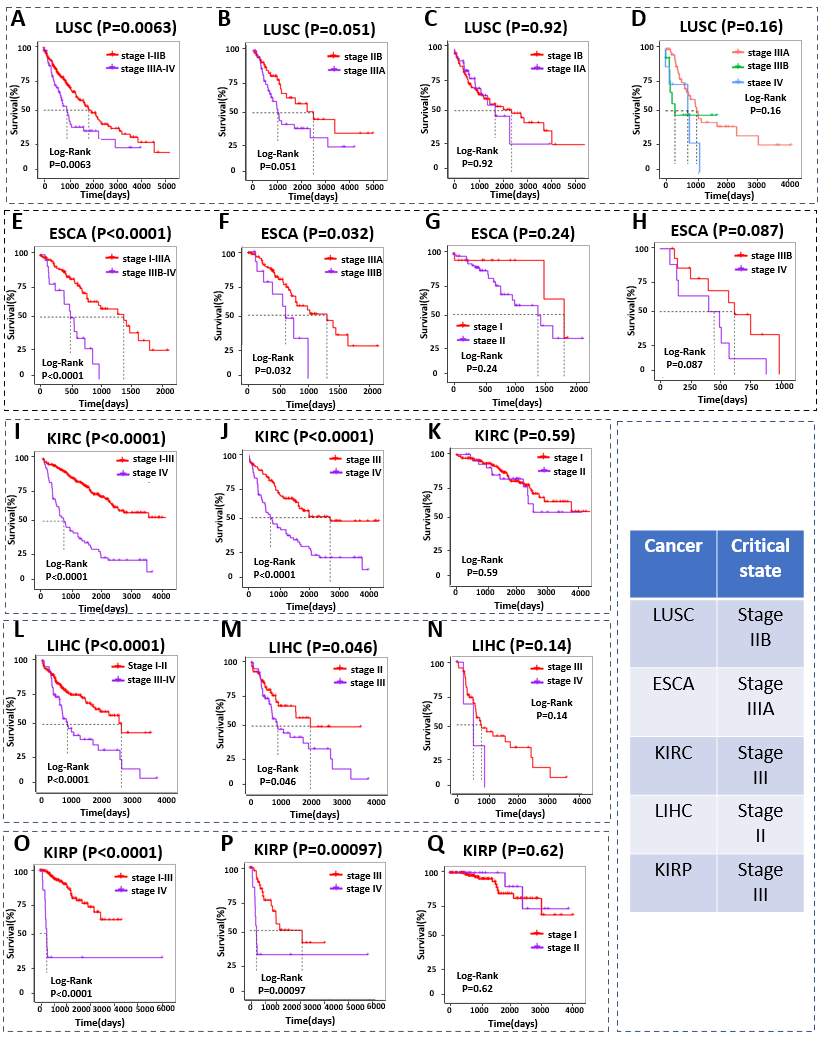


**Figure S6: |** **Validating the identified critical state of LUSC, ESCA, KIRC, LIHC, and KIRP.**

Comparison of survival curves before and after critical state for five cancers: LUSC, ESCA, KIRC, LIHC, and KIRP. Before the identified critical stage, there is generally a high expectation of life after diagnosis, while after the critical stage, there is a much lower expectation of survival after diagnosis. However, before and after any other stages, there was no significant difference in the prognosis.
