# Supplementary material for: Identifying the critical states and dynamic network biomarkers of cancers based on network entropy
Source: J Transl Med. 2022 Jun 6;20:254. doi: 10.1186/s12967-022-03445-0 (PMC9172070; doi:10.1186/s12967-022-03445-0)
Supplement: Supplementary file 2 — Additional file 2: Table S1. The number of tumor samples within each stage in the cancer dataset from TCGA. [file 12967_2022_3445_MOESM2_ESM.docx]

Table S1. The number of tumor samples within each stage in the cancer dataset from TCGA.

|  | TA samples | Stage I | | Stage II | | Stage III | | | Stage IV |
| --- | --- | --- | --- | --- | --- | --- | --- | --- | --- |
|  |  | Stage  IA | Stage IB | Stage IIA | Stage IIB | Stage IIIA | Stage IIIB | Stage  IIIC |  |
| LUSC | 49 | 65 | 112 | 55 | 63 | 48 | 14 | 0 | 7 |
| STAD | 32 | 53 | | 62 | 49 | 63 | 52 | 35 | 38 |
| LUAD | 58 | 230 | | 39 | 59 | 62 | 10 | 0 | 21 |
| COAD | 41 | 80 | | 173 | 13 | 28 | 60 | 44 | 66 |
| READ | 10 | 30 | | 50 | | 12 | 25 | 13 | 24 |
| ESCA | 11 | 16 | | 69 | | 35 | 14 | 0 | 8 |
| KIRC | 73 | 197 | | 41 | | 112 | | | 68 |
| LIHC | 50 | 157 | | 78 | | 70 | | | 5 |
| THCA | 33 | 218 | | 44 | | 82 | | | 37 |
| KIRP | 33 | 179 | | 25 | | 51 | | | 15 |

TA samples: tumor-adjacent samples
