# Supplementary material for: Identifying the critical states and dynamic network biomarkers of cancers based on network entropy
Source: J Transl Med. 2022 Jun 6;20:254. doi: 10.1186/s12967-022-03445-0 (PMC9172070; doi:10.1186/s12967-022-03445-0)
Supplement: Supplementary file 3 — Additional file 3: Table S2. The optimistic LNE (O-LNE) biomarkers and pessimistic LNE (P-LNE) biomarkers in KIRC, LUSC, STAD, LIHC, LUAD, ESCA, COAD, READ, THCA, and KIRP. [file 12967_2022_3445_MOESM3_ESM.docx]

Table S2: The optimistic LNE (O-LNE) biomarkers and pessimistic LNE (P-LNE) biomarkers in KIRC, LUSC, STAD, LIHC, LUAD, ESCA, COAD, READ, THCA, and KIRP.

| KIRC | | LUSC | | STAD | | LIHC | | LUAD | |
| --- | --- | --- | --- | --- | --- | --- | --- | --- | --- |
| OHLE | **PHLE** | **OHLE** | **PHLE** | **OHLE** | **PHLE** | **OHLE** | **PHLE** | **OHLE** | **PHLE** |
| CLIP4  PGD  TIE1 | CDCP1  EPB41  IGF2R  PPIE  S100A11  SKIV2L | ACP1  ERH  FGF11 | AHNAK  EIF3B  PABPC1L  PLAU | CATSPERB  TIMM8A  ZNF350 | ACE2  SRI | ATP2B4  CYP2A6  CD81  FCN2  IRF6  RAMP2  RGS5 | ENO1  G6PD  HMGA1  MYBL2  SEC61A1  TKT  TSEN34  TTK | ENO3  HKDC1  IL4I1  IMPA2  KIF2A  SMARCC1 | INTS1  ITGB4  PRSS3  RIMS2 |

| ESCA | | COAD | | READ | | THCA | | KIRP | |
| --- | --- | --- | --- | --- | --- | --- | --- | --- | --- |
| OHLE | **PHLE** | **OHLE** | **PHLE** | **OHLE** | **PHLE** | **OHLE** | **PHLE** | **OHLE** | **PHLE** |
| APOBEC3G | HSPA1B  IGFBP7  PFN2  SPP1 | NOX1  TMED2  TNNT1 | IGFBP3  POLM  UBE2I | DSN1  PSAP  TRMT6 | PLOD1  TSTA3 | ANXA1  ERBB3  GRB7  LLGL1 | DDIT4  MYH9  NACAD  SEMA3C  SOX18  TMEM30A  VARS2 | NOX4  PLD3 | PABPC1  MRPL51 |

Red color: *p*-value < 0.01

Other color: 0.01 < *p*-value <0.05
